# Even with time, conflict adaptation is not made of expectancies

**DOI:** 10.3389/fpsyg.2014.01042

**Published:** 2014-09-16

**Authors:** Luis Jiménez, Amavia Méndez

**Affiliations:** Facultad de Psicología, Universidad de Santiago de CompostelaSantiago de Compostela, Spain

**Keywords:** conflict adaptation, stroop task, expectancies, congruency sequence effect, cognitive control, reactive control

## Abstract

In conflict tasks, congruency effects are modulated by the sequence of preceding trials. This modulation has been interpreted as a strategic reconfiguration of cognitive control, depending on the amount of conflict encountered on the very last trial, and occurring unconditionally whenever there is time to produce it (Notebaert et al., 2006). Jiménez and Méndez (2013) arranged a 4-choice Stroop task with a response-to-stimulus interval (RSI) of 0 ms, and they found that, under these conditions, congruency effects may become dissociated from the explicit expectancies assessed over analogous, but independent, trials. The present study generalizes this phenomenon to a condition with larger RSI, and it shows that participants’ performance does not rely on expectancies unless the task includes a specific requirement to generate and report on these expectancies. The results are interpreted as providing new insights with respect to the status of conflict adaptation effects.

## INTRODUCTION

Is *conflict adaptation* an illusion? The response to this question may depend on the meaning of the italicized expression. If it is taken as a descriptive label, referring to the fact that congruency effects are adaptively modulated by the congruency of the previous trials, then we will argue that this is not an illusion, but rather it is a pervasive phenomenon resulting from the highly dynamic and adaptive nature of the cognitive system, which always takes advantage of its previous experience to process and respond to the upcoming events in ways similar to those practiced in the past. If, on the other hand, conflict adaptation is strictly defined as the result of a top–down expectancy, or as a strategic reconfiguration of the cognitive control system depending on the conflict encountered on the very last trial, and occurring unconditionally whenever there is time to produce it, i.e., as a result of a control process which takes about 200 ms to complete, as suggested by Notebaert et al. (2006), then we will contend that this is more an exception than the rule, and that this kind of process is only activated under very specific conditions.

Cognitive conflict arises whenever there are two features in a display which are potentially incongruent with each other. In the traditional Stroop task, for instance, participants are told to respond to the color in which a word is written, and congruency effects refer to the fact that people respond faster when the color is congruent with the meaning of that word (e.g., “GREEN” written in green), than when they are incongruent with each other (e.g., “GREEN” written in red). The most popular way to analyze conflict adaptation in this context has been to look at the “congruency sequence effect” (CSE), which arises as a difference in the effect of congruency depending on the congruency of the previous trial. As a rule, the effect of congruency tends to increase after a congruent trial, and it tends to decrease after an incongruent trial. However, in those experiments using fewer than four different stimuli and responses, it becomes problematic to distinguish between the alleged effects of conflict adaptation and those potentially caused by episodic memory factors, such as the immediate repetition of a trial (Mayr et al., 2003), or the repetition of a feature that reappears immediately in a different role (i.e., a target feature reappearing as a distractor, or vice versa, Hommel et al., 2004).

To avoid both total and partial repetitions, Jiménez and Méndez (2013) arranged a 4-choice Stroop task, and they grouped the four possible colors in two alternating pairs, so that trials displaying the word “RED” or “GREEN” printed in red or green, alternated with trials showing the word “BLUE” or “YELLOW” printed in blue or yellow. These conditions, which are structurally analogous to those used in some variants of the flanker task (Mayr et al., 2003) or of the prime-probe arrow task (Kim and Cho, 2014; Schmidt and Weissman, 2014), served not only to avoid immediate feature repetitions, but also to maintain a relatively high proportion of congruent trials (50%), without associating the distractors more often with the congruent response than with any of the possible incongruent responses (see Schmidt and De Houwer, 2011 or Mordkoff, 2012, for discussions about how contingency learning may get confounded with the CSE in congruency tasks). In order to reduce the potential effects of explicit expectancies, Jiménez and Méndez (2013) set the response-to-stimulus interval (RSI) to 0 ms, and they found that the CSE disappeared under these conditions, at least when it was measured in the standard way, as the impact of the congruency of the last trial on the congruency effect measured on the following trial. However, when the congruency of a larger set of previous trials was taken into account, they obtained a significant linear trend, showing that the effect of congruency was inversely proportional to the amount of conflict accumulated over the last few trials. Thus, the effect became maximal after a run of three consecutive congruent trials (C,C,C,-), but it decreased progressively after runs of two (I,C,C,-) or just one (x,I,C,-) previous congruent trial, and it decreased further after runs of one (x,C,I,-), two (C,I,I,-), or three (I,I,I,-) consecutive incongruent trials ^1^.

Interestingly, the linear pattern observed in the measures of reaction time (RT) from Jiménez and Méndez (2013) could not be explained in terms of the explicit expectancies developed over those larger contexts, since those expectancies were measured independently over different blocks, and they revealed the development of a bias opposite to the effects observed in the measures of RT. According to the gambler’s fallacy (Jarvik, 1951), participants’ expectancies were biased to predict an incongruent successor after a series of two or more consecutive congruent trials, whereas the RT measured in those low-conflict contexts showed the largest advantage in favor of responding to these supposedly “unexpected” congruent trials. Reciprocally, after a series of two or more incongruent trials, participants reported to be expecting a change to a congruent successor, but these high-conflict contexts resulted in the minimal difference in RT between responding to a congruent and to an incongruent successor.

The dissociation found in Jiménez and Méndez (2013) between explicit expectancies and long-range conflict adaptation effects was interpreted by the authors as indicating that explicit predictions would not be affecting performance in speeded conditions, but that the observed adaptation effects would reflect an *inertial* adaptation to the amount of conflict (or lack of conflict) experienced over the last few trials, which would improve responding to those trials which make analogous control demands to those made by the series of previous trials (see also Lamers and Roelofs, 2011; Schlaghecken and Martini, 2012, for similar conclusions) ^2^. However, given that this pattern of results had been obtained in conditions which minimized the chances of developing and exploiting any explicit prediction, and in which those expectancies were measured over independent blocks, differing widely in their temporal arrangement with respect to the regular Stroop blocks, we set to conceptually replicate these results under temporal conditions which may leave enough room for strategic processes to operate. According to Notebaert et al. (2006), an RSI of 200 ms might be enough to produce a top-down reconfiguration of the control system. However, because recent parametric studies have documented that the CSEs are usually larger with RSI between 500 and 1000 ms (Egner et al., 2010; Duthoo et al., 2014) we decided to set a fixed RSI of 750 ms, that could be long enough to allow for the development of strategic operations, but not so long as to dilute the effects of the series of previous trials.

## MATERIALS AND METHODS

The experiment was conducted in accordance with the Spanish regulations on behavioral research. Eighteen students of psychology from the University of Santiago de Compostela participated in the experiment in exchange for a monetary fee. The procedure closely followed that of Experiment 1 from Jiménez and Méndez (2013), with the exception that the RSI was fixed at 750 ms. The task required participants to respond to the color of a word that might be written in red, blue, yellow, and green, by using, respectively, the keys corresponding to the letters “z,” “x,” “n,” and “m,” which were covered by congruently colored stickers. Responses were emitted using the index and middle fingers of both hands. After a short practice block in which participants got familiar with the mapping between colors and keys, using words unrelated to the colors, participants completed five experimental blocks with Stroop stimuli, consisting of the Spanish words for red (*rojo*), blue (*azul*), yellow (*amarillo*), and green (*verde*). Participants were instructed to ignore the meaning of those words, and to respond exclusively on their color. They were also informed that the color could be congruent with the word meaning in approximately a half of the trials, but that color and word meaning would be incongruent with each other in the other half of trials. Errors were explicitly marked by a tone, and the stimulus remained on the screen until the correct key was pressed. The next trial arose after an RSI of 750 ms, composed of a fixation point appearing at the center of the screen for 500 ms, and a blank interval of 250 ms which preceded the next word. At the end of each block, participants were informed about the percentage of correct responses produced over the last block, and they were asked to keep responding as fast as possible, while maintaining the level of errors below 10%.

To avoid color repetitions over successive trials, the four colors were grouped into alternating pairs, producing an alternation between trials showing the word “RED” or “GREEN” printed in red or in green, and trials showing the word “BLUE” or “YELLOW” printed in blue or in yellow. The colors grouped into a target/distractor pair were selected so that their responses were assigned to different hands, thus avoiding that the alternating color pattern would amount to a pattern of alternating hands (cf. Kim and Cho, 2014, Experiment 2). Each block contained 176 trials, including exactly 88 congruent and 88 incongruent trials. The runs of trials were also controlled so as to conform to those expected by chance (see Perruchet et al., 2006). Thus, we included as many runs of a single congruent trial (16) or of a single incongruent trial (16), as there were runs of two consecutively congruent trials (16) or of two consecutively incongruent trials (16). From here on, because chance probabilities of producing larger runs should be multiplicatively smaller than those of their smaller components, we included 8 runs of three consecutively congruent trials, 8 runs of three consecutively incongruent trials, four runs of four consecutively congruent trials, and 4 runs of four consecutively incongruent trials. The probability of producing still larger runs by chance was too low to permit a reliable measure of their effects, and therefore we set length-four as the maximum length of trials of the same type. All these runs were randomly intermixed for each block and participant, with the constraint that congruent and incongruent runs should alternate with each other, so as to avoid producing larger runs out of the concatenation of shorter ones. As a consequence of this specific design, not only the proportion of congruent and incongruent trials, but also the conditional probabilities of finding repetitions or alternations of congruency were balanced for each individual block.

Blocks 1, 3, and 4 were arranged as standard Stroop blocks. In blocks 2 and 5 participants also responded to regular Stroop trials, but after each of these trials they were presented with an explicit measure of their expectancies. At the beginning of each of these Expectancy blocks, participants were awarded with 100 points, and they were asked to bet 3, 2, or 1 of their points depending on the certainty with which they could predict that the next trial was going to be congruent or incongruent: (3) “sure,” (2) “fairly sure,” or (1) “guessing.” Participants reported their bets verbally, and the corresponding score was entered by the experimenter manually using a second keyboard. The next trial appeared 750 ms after the bet was entered, including an update of their remaining points, together with the next Stroop trial, which also served as an indirect feedback on the accuracy of the previous prediction.

## RESULTS

Jiménez and Méndez (2013) analyzed RT exclusively over the regular Stroop blocks, mainly because in their Expectancy blocks the predictions were entered by the participants with the aid of a computer mouse, which forced them to continually shift their right hand from the keyboard to the mouse, and then back to the keyboard. In the Expectancy blocks from the present experiment we replicated the original procedure with two exceptions: participants reported their bets verbally, while maintaining their fingers on the response keys, and the encoding of each prediction was entered in the computer by the experimenter, and was followed by the next Stroop trial after an RSI of 750 ms, just as during the standard Stroop task. In this way, even though our main interest was still focused on the analysis of the CSE observed during the standard Stroop blocks, we were also able to explore the effects of congruency that might be observed on the Expectancy blocks. Duthoo et al. (2013) reported that a close association between expectancies and congruency effects was obtained when both measures were taken on the very same trials. Thus, an additional objective of this study was to ascertain whether this association between expectancies and congruency effects could be extended to the regular Stroop trials when participants have enough time to develop a prediction, or whether such association could be rather restricted to conditions in which expectancies were generated in response to explicit task demands.

Alpha level was set at 0.05 for all reported analyses. Wherever there was a risk of violation of the sphericity assumption, we relied on the Greenhouse–Geisser ε-corrected *p* values, but we reported the nominal degrees of freedom for simplicity. We will restrict the report to the analyses of RT, but we confirmed in each case that the reported effects could not be explained in terms of a trade-off between speed and accuracy. In the Stroop blocks, we analyzed the effect of congruency, the first-order (i.e., standard) CSE, and the progressive CSE, defined as the progressive changes in the effect of congruency which depended on the type and length of the last run of trials ^3^. As for the Expectancy blocks, we assessed participants’ expectancies in the contexts defined by the same runs of trials, and we also analyzed whether these expectancies were consistent with their speeded performance, either during the regular Stroop blocks, or during the same blocks in which the expectancies were measured.

### STROOP BLOCKS

**FIGURE 1:**
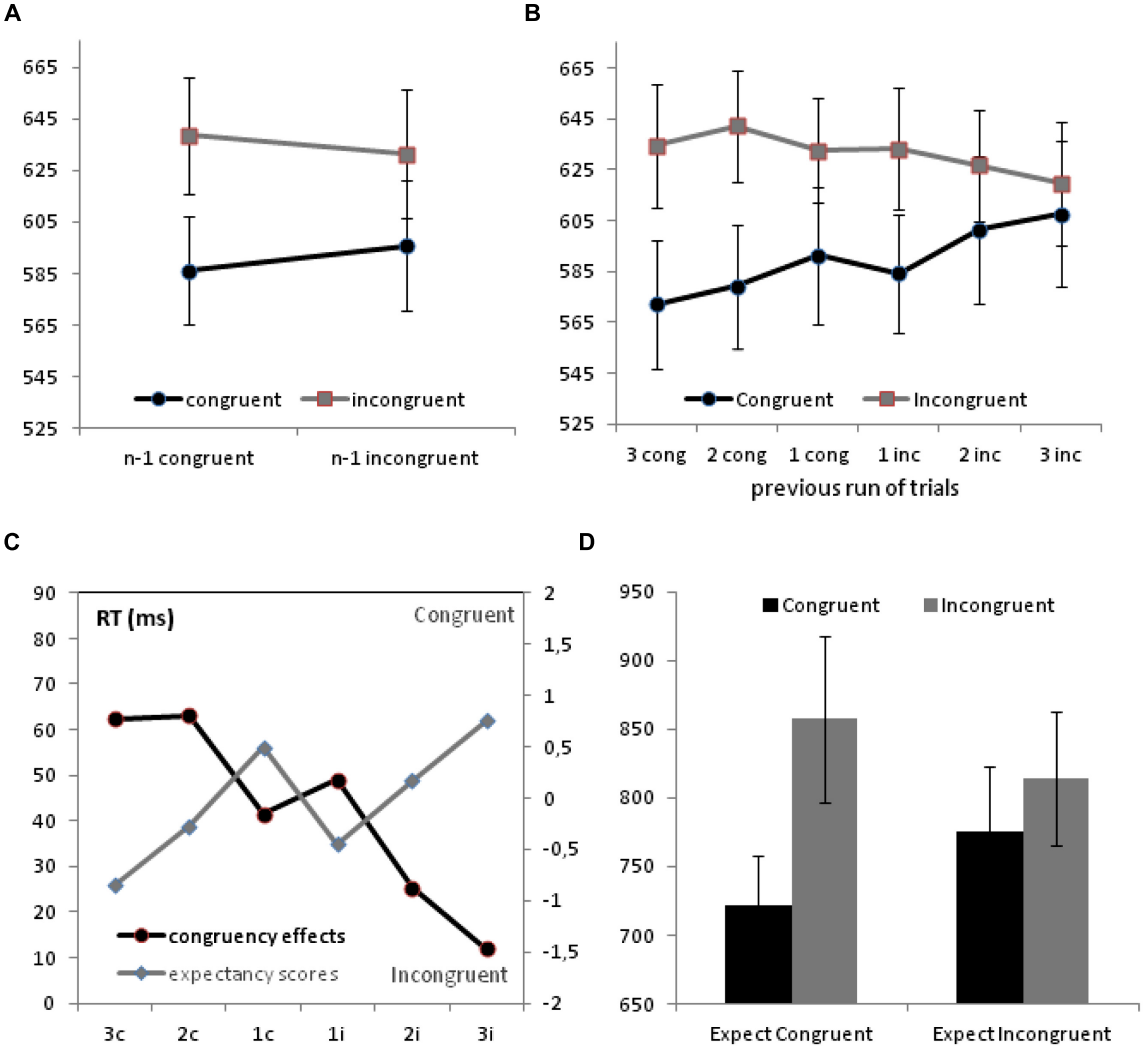
**(A)** Congruency sequence effects (CSE). **(B)** Progressive variation of the congruency effects depending on the type and length of the previous run of trials. **(C)** Dissociation between the Congruency effects as taken from the standard Stroop blocks (left axis) and the expectancy scores taken from the Expectancy blocks (right axis). **(D)** Congruency effects as measured on the Expectancy blocks, represented separately for trials in which participants expected a congruent or an incongruent successor.

As in Jiménez and Méndez (2013), we excluded the first trial from each block, those trials containing an error, and the trial immediately following an error, as well as outliers, defined as those trials with RT straying more than 3 standard deviations from each block and individual mean. In total, 6.9% of the trials from the three Stroop blocks were excluded by applying these criteria. Data from these three blocks were collapsed together, in order to produce a sufficient number of observations even for larger contexts. The measure of RT was submitted to an analysis of variance (ANOVA) with Congruency (2) and Previous Congruency (2) as repeated factors. This analysis showed a strong effect of Congruency, *F*(1,17) = 36.30, *p* < 0.001; η_p_^2^ = 0.68, but not a main effect of Previous Congruency (*F* < 1). A significant Congruency × Previous Congruency interaction, *F*(1,17) = 5.17, *p* = 0.036; η_p_^2^ = 0.23, indicated that the Stroop effect was significantly larger after a congruent trial (52 ms) than after an incongruent trial (36 ms, see **Figure 1A**). Thus, the standard CSE, which had not been obtained in the original experiment by Jiménez and Méndez with an RSI of 0 ms, was observed in this case by using a larger RSI, and in conditions in which these sequential effects were properly distinguished from the potential influence of feature repetitions (cf. Mayr et al., 2003; Hommel et al., 2004), as well as from contingency learning confounds (Schmidt and De Houwer, 2011; Schmidt and Weissman, 2014).

The analysis of the progressive variation of these congruency effects depending on the type and length of the previous run of trials was conducted using context (6: runs of 3, 2, or 1 congruent trials, and runs of 1, 2, and 3 incongruent trials) and Congruency (2) as repeated factors. Again, this analysis showed a robust effect of Congruency, *F*(1,17) = 28.10, *p* < 0.001; η_p_^2^ = 0.62, but not an effect of context (*F* < 1). The Congruency × Context interaction was also significant in this analysis, *F*(5,85) = 3.35, *p* = 0.02; η_p_^2^ = 0.17, and it was qualified by a significant linear contrast, *F*(1,17) = 10.40, *p* = 0.005; η_p_^2^ = 0.38, which confirmed that the effect of congruency was inversely proportional to the amount of conflict accumulated over the last few trials. Thus, it reached larger values in the context of previous congruent trials (62, 63, and 41 ms, respectively, after runs of 3, 2, or 1 previous congruent trial), and decreased to values of 49, 25, and 12 ms in the contexts defined by 1, 2, or 3 consecutive incongruent trials (see **Figure 1B**). This pattern of results was largely consistent with that found in Jiménez and Méndez (2013), and it showed that the conflict adaptation effect did not arise immediately after a single trial of each type. Thus, even though we obtained a significant CSE when we assessed the effect in the standard way, it is important to notice that this sequential effect depended on the accumulation of several previous trials of the same type, and it was not observed if we remove the impact of larger runs and compare specifically the congruency effect provoked by a single congruent trial (x,I,C,-), with that obtained after a single incongruent trial (x,C,I,-). In this case, the effect of congruency was numerically smaller after a congruent trial (41 ms) than after an incongruent trial (49 ms).

### EXPECTANCY BLOCKS

Participants’ expectancies were coded for each trial as in Jiménez and Méndez (2013), by scoring 1, 2, or 3 points for the respective bets in favor of a congruent successor, and by changing the sign to -1, -2, or -3 for the corresponding bets made in favor of an incongruent successor. We collapsed those values over the two expectancy blocks, and conducted an ANOVA on these scores with context (6) as a single repeated factor. The analysis showed that the expectancy scores were significantly affected by the Context, *F*(5,85) = 6.35, *p* < 0.01; η_p_^2^ = 0.27. As predicted by a repetition expectancy account, participants predicted a congruent successor after a single congruent trial (0.50), and an incongruent successor after a single incongruent trial (-0.44). These two predictions were significantly different from each other, *t*(17) = 3.68, *p* = 0.002. In contrast, after a longer series composed of two or three trials of the same type, participants predicted an alternation pattern, as if they fell into the gambler’s fallacy. Specifically, after a row of three congruent trials participants predicted an incongruent successor (-0.85), whereas after a similar row of three incongruent trials they reported to expect a congruent successor (0.76). These two predictions were also significantly different from each other, *t*(17) = 3.15, *p* = 0.006.

The compensation bias arising in the expectancy scores replicated the results reported in Jiménez and Méndez (2013), and it stood in sharp contrast with the RT distribution obtained over the regular Stroop blocks. **Figure 1C** represents the congruency effects observed on the relevant contexts from the standard Stroop blocks, together with the expectancy measures taken over analogous contexts from the Expectancy blocks. As it can be observed, the figure shows a striking dissociation between both measures, showing larger increases of the effect of congruency precisely in those contexts in which participants declare to be expecting an incongruent successor (i.e., after runs of two or three consecutive congruent trials), and showing larger decreases of this effect in those contexts in which participants reported to be expecting a congruent successor. This pattern is not compatible with the claim that explicit expectancies directly modulate the effect of congruency in the standard Stroop task, and therefore indicates that having enough time to elicit a prediction is not enough for participants to generate those predictions, and to rely on them in the context of a standard Stroop task.

In the face of the dissociation observed between congruency effects and expectancy scores when they are gathered from analogous contexts, but out of different blocks of trials, one may wonder whether a similar dissociation could also be obtained when one looks at both effects strictly at the same moment. The results from Duthoo et al. (2013) indicated that expectancies and congruency effects were closely associated when both measures were taken on the very same trials. Thus, to assess whether a similar association could arise over the Expectancy blocks from the present experiment, we classified each trial from the Expectancy blocks in two different categories, depending on whether the participants declared to be expecting a congruent or an incongruent successor, and we looked at the effects of congruency observed in those trials depending on these explicit expectancies. An ANOVA conducted on the RT from the Expectancy blocks with declared Expectancy (2) and Congruency (2) as two repeated factors, showed no significant effect of Expectancy, *F*(1,17) = 1.22, *p* = 0.29, but it showed a significant effect of Congruency, *F*(1,17) = 22.95, *p* < 0.001; η_p_^2^ = 0.58, and a significant Expectancy × Congruency interaction, *F*(1,17) = 11.38, *p* = 0.004; η_p_^2^ = 0.40. As shown in **Figure 1D**, these results indicate that, in the Expectancy blocks in which the participants were required to generate a prediction, the effect of congruency was indeed associated with those explicit predictions: even though the effect of congruency was significant even in those trials in which the participants declared to be expecting an incongruent successor (775 vs. 814 ms), *t*(17) = 3.48, *p* = 0.003, the effect was much larger when they reported to be expecting a congruent successor (722 vs. 857 ms).

## DISCUSSION

This study replicated a previous dissociation reported in Jiménez and Méndez (2013) between explicit expectancies and conflict adaptation effects, in conditions in which the effects of total and partial repetitions were controlled (cf. Mayr et al., 2003; Hommel et al., 2004), and in which the measures of expectancies and the effects of conflict adaptation were assessed independently over different sets of Stroop blocks. The results indicate that the dissociation originally obtained using an RSI of 0 ms can also be extended to less restrictive temporal conditions, and particularly that it can be generalized to an RSI of 750 ms, an interval which should leave plenty of time for any potential strategic adjustment to take place (cf. Notebaert et al., 2006). Under these conditions, the explicit predictions elicited in the context of the Expectancy blocks reflect a compensation bias, which tends to predict a change after a series of two or more trials of the same congruency type. Thus, participants report to be expecting an incongruent successor after a run of two or more congruent trials, and a congruent successor after a series of two or more incongruent trials. In contrast, the effect of congruency, as measured over the standard Stroop blocks, shows an opposite pattern that decreases progressively with the accumulation of conflict, thus growing to their maximal scores after a series of consecutive congruent trials, and decreasing to their minimal values after a series of consecutive incongruent trials. Thus, in sharp contrast to any expectancy account of the CSE, these results indicate that the effect of congruency decreases precisely in those contexts in which the participants report to be expecting a congruent successor, and reaches its maximal levels in those contexts in which participants report to be expecting an incongruent successor.

Interestingly, the dissociation observed between congruency and expectancy scores when both measures were taken from independent blocks was no longer maintained when they were taken from the very same trials, that is, when the congruency effects were computed in a context that explicitly required participants to elicit an explicit expectancy on each trial. In those conditions, which resembled those arranged by Duthoo et al. (2013), we found an association between expectancies and congruency effects, indicating that the reported expectancies were indeed efficacious to modulate RT when they were actually elicited in response to an expectancy test. Thus, the failure to obtain a direct effect of the expectancies over the standard Stroop blocks, together with the observation that this effect exists in the expectancy blocks, could be taken to indicate that such explicit expectancies are not built by default, even if there is enough time available to do that, but that they may be generated on request, and of course in that case they affect performance.

The most important discrepancy between the results reported by Duthoo et al. (2013) and those obtained in the present experiment, and in the previous experiments by Jiménez and Méndez (2013), refers to the specific pattern of expectancies observed in each paradigm. Thus, whereas Duthoo et al. (2013) reported that their participants kept predicting repetitions even after a series of two or three trials of the same type, according to what they dubbed as “the hot-hand fallacy,” our participants showed a less extended propensity to predict repetitions, and they felt quickly into the opposite “gambler’s fallacy.” We can only speculate about the possible source of this empirical difference, but we surmise that the use of just two colors in the case of the study by Duthoo et al. (2013) instead of the four different colors arranged in our paradigm, may be partially responsible of producing a difference in the perceived likelihood of repetitions, and may ultimately affect their expectancy scores. In any case, our results did closely replicate the dissociation pattern first reported by Jiménez and Méndez (2013), and they confirmed that such dissociation is not exclusive of those speeded conditions which leave no room for top–down preparation, and that the effect of explicit predictions may be absent unless they are explicitly requested.

Thus, coming back to the original question underlying this research topic, about the status of conflict adaptation effects, we would like to conclude by suggesting that many of the so-called “reactive” conflict adaptation effects, rather than being exerted by some “shadow” set of control mechanisms, which exert the same functions as those fulfilled by the proactive mechanisms, but only in a faster, more automatic, and less conscious mode, could be better explained as the integrated outcome of a mixture of mechanisms which may have not been designed specifically for such control purposes, but which take advantage of the system’s past experience to respond to new events in ways similar to those which were proven effective in the past. From this point view, finally, these mechanisms, which may comprise those processes underlying phenomena such as perceptual priming, process priming, episodic memory, implicit contingency learning, temporal learning, and the like, perhaps should not be taken as potential confounds, or as alternatives to the genuine “mechanism” of reactive control. Rather, they could just be considered as components of the cognitive toolbox which, together, implement this delicately adaptive, automatic, complex, and highly dynamic, function of control.

## Conflict of Interest Statement

The authors declare that the research was conducted in the absence of any commercial or financial relationships that could be construed as a potential conflict of interest.

## Footnotes

1 A run of trials is defined backward, as the maximum number of consecutive trials from a previous context which belong to a given congruency class. For instance, a run of two congruent trials (I,C,C,-) is defined as the context in which trials n-1 and n-2 are both congruent, but trial n-3 is incongruent. A shorter run of just one congruent trial (x,I,C,-) is defined as a context in which trial n-1 is congruent (C) but trial n-2 is incongruent (I). In that case, the nature of trial n-3 becomes irrelevant (x).

2 In this context, we will refer to the pattern obtained by Jiménez and Méndez (2013) as a conceptual “dissociation” between expectancies and conflict adaptation effects, even though in statistical terms their results showed a negative association, rather than a statistical dissociation. Given that it is conceptually very implausible that expecting a congruent successor could directly result in a slower RT to these expected trials, the conclusion inferred from that negative association was that expectancies and CSE should be driven by independent factors: whereas the CSE could reflect an inertial adaptation to the amount of conflict experienced over the last few trials, expectancies would be built exclusively when they are explicitly required, and they would be modulated by some sort of anti-inertial, or compensating bias, resembling the gambler’s fallacy.

3 Regardless of the nominal “runs” of trials introduced by design to generate a balanced sequence, these analyses were conducted considering all the trials satisfying the criteria for a given type and length of context. Thus, for instance, the second congruent trial from a nominal run of three consecutive congruent trials (C,C,C,-) was not only the context for a forthcoming successor, but also the successor of a shorter context.
